# Comparative Analysis of Leukocyte Proportions and Morphology in Wild Muridae and Arvicolinae Rodents

**DOI:** 10.3390/biology15171528

**Published:** 2026-09-03

**Authors:** Eleni Rekouti, Eleftheria E. Rosmaraki, George P. Mitsainas

**Affiliations:** 1 Section of Animal Biology, Department of Biology, University of Patras, GR-26504 Patras, Greece; 2 Section of Genetics, Cell & Developmental Biology, Department of Biology, University of Patras, GR-26504 Patras, Greece; rosmaraki@upatras.gr

**Keywords:** leukocyte profile, *Apodemus*, *Microtus*, *Clethrionomys*, azurocytes, wildlife rodents

## Abstract

Hematological profiles of wild rodents provide important insights into their species-specific adaptations, physiological status, and immune responses. However, reference data for wild species remain limited since these are mainly derived from laboratory rodents. This study investigated the leukocyte composition and morphology in 113 wild rodents representing the family Muridae and the subfamily Arvicolinae from several regions of seven prefectures of Greece. Blood samples were collected from live-trapped individuals, and peripheral blood smears were analyzed using Giemsa staining. We evaluated leukocyte variation related to taxonomy and sex. Marked differences were observed between Muridae and Arvicolinae, while lymphocytes predominated in both. Arvicolinae exhibited higher neutrophil and lower lymphocyte proportions than Muridae. Leukocyte profiles were relatively consistent among Arvicolinae, but interspecific variation was evident within the genus *Apodemus*. No statistically significant differences in leukocyte proportions were detected between males and females. Morphologically, reactive lymphocytes were observed in Muridae, while cells morphologically consistent with azurocytes were observed in *Microtus thomasi* and in the single examined individuals of *M. felteni* and *M. subterraneus*. The findings provide new comparative hematological data for wild rodents and highlight the importance of species-specific reference values in eco-immunological studies.

## Simple Summary

Wild rodents play a crucial role in our ecosystems and are frequently studied to understand how animals adapt to their environments. While much is known about the immune systems of laboratory rodents, data for wild populations—which face a variety of natural stressors—remain limited. In this study, we examined the blood cell profiles of several wild rodent species found in Greece, dwelling either above or below ground, to establish baseline physiological data. We found differences in leukocyte distribution among the examined rodent groups and species, providing new comparative information on their hematological profiles. Notably, we observed cells morphologically consistent with azurocytes in three *Microtus* species, in which such cells have not previously been reported. These findings provide important reference data for researchers studying wildlife. By understanding how the immune systems of these rodents function and vary in the wild, we gain deeper insight into how different species cope in nature with environmental challenges, thus increasing their value as indicators of ecosystem health.

## 1. Introduction

For decades the immunological responses of rodents have been under scientific scrutiny. Although numerous studies have focused on laboratory mice and rats, extrapolating these findings to wild populations remains challenging, as wild individuals are constantly exposed to fluctuating environmental factors that strongly modulate immunological responses [1,2]. Despite these complexities, systematic research is conducted to establish the immune profiles of wild species by characterizing key immunological markers, such as cytokines, specialized cellular receptors, and transcription factors [2,3,4,5,6,7,8,9].

In wild rodents, hematological indicators are highly dynamic and susceptible to a wide range of exogenous and endogenous factors. Specifically, individual traits (such as age, reproductive status and overall physical condition), ecological traits (including seasonality and population density), and external factors (such as transient or chronic stressors, infections by pathogens, and anthropogenic effects) have all been shown to significantly alter these hematological indices [5,10,11,12,13,14,15].

Advanced techniques routinely used to determine the immunophenotype of leukocytes in laboratory rodents, such as flow cytometry, are often inapplicable to wild species. Commercially available monoclonal antibodies (mAbs) targeting specific clusters of differentiation (CD) markers are optimized for laboratory strains and frequently fail to exhibit cross-reactivity with the leukocyte surface receptors of wild individuals. Since developing species-specific markers is both costly and time consuming [5,16], researchers must frequently rely on alternative approaches—such as classical morphological evaluation of blood smears—to accurately discriminate and assess leukocyte types [16]. Blood smear examination provides information on both the relative proportions and morphological characteristics of leukocytes, and it often serves as an important complementary method to automated complete blood count (CBC), which can accurately determine the proportions of whole blood cell populations in a sample [17].

White blood cells (WBCs) are fundamental to both innate and adaptive immunity. The innate, first-line defense is primarily mediated by neutrophils, monocytes, eosinophils, basophils, and Natural Killer (NK) cells, whereas B and T lymphocytes drive the adaptive immune response [18]. Beyond these typical morphological forms, specific lymphocyte subtypes are often encountered in peripheral blood, activated during particular immune responses. Notable among those are reactive lymphocytes (RLs) or immunocytes, which are present in small proportions in peripheral blood in various vertebrate taxa, such as mammals, birds, reptiles, and fish [19,20,21], and proliferate following antigenic stimulation. RLs can originate from immunoglobulin-producing B lymphocytes [21,22] or activated T lymphocytes, commonly known as Downey cells [23]. Morphologically, they are distinguished from normal lymphocytes by their larger size and an increased volume of intensely dark blue-stained cytoplasm [20]. In addition, their nuclei may not have a typical spherical shape but instead exhibit irregular (bilobed or amoeboid) morphology [21,22].

A particularly distinct leukocyte type, functionally analogous to NK cells, is the azurocyte (AZ). First described in 1987 in *Microtus pennsylvanicus*, AZs are notably larger than normal lymphocytes and derive their name from the numerous large, azurophilic granules within their cytoplasm [24]. In immature individuals and non-reproductive adults AZs occur at very low concentrations (<1%) in peripheral blood. However, their abundance increases dramatically during the reproductive phase (April–October), mainly in pregnant or lactating females, driven by elevated progesterone levels [24,25]. It has been proposed that AZs represent a lineage-specific type of NK cell in *Microtus* voles [26] that may be important for reproduction in pregnant *Microtus* females and may help trigger abortion when conditions are unfavorable [25]. Until now, confirmed AZ records are restricted to the peripheral blood of some *Microtus* species, namely, *M. pennsylvanicus* [24], *M. townsendii* [24], *M. oeconomus* [24,27], *M. agrestis* [25], *M. arvalis* [28] and *M. rossiaemeridionalis* [28]. While granular lymphocytes with a similar morphology have been reported in *Clethrionomys glareolus*, their definitive classification requires further histochemical or immunological validation [29,30].

Due to their status as the primary sources of laboratory mammal strains, *Mus musculus* and *Rattus norvegicus* are the most well-studied rodent species with regard to leukocyte profiles. Under homeostatic conditions, lymphocytes constitute the predominant leukocyte type (ca. 70–75%), followed by neutrophils (ca. 20–25%). Monocytes account for 2–6% in mice and 3–11% in rats, whereas eosinophils (0–3% and 0.1–4.8%, respectively) and basophils (<1% and rats <2%, respectively) are the least frequently encountered WBCs in peripheral blood. Both Large Granular Lymphocytes (LGLs) and RLs have also been reported in their blood smears [31]. While leukocyte proportions in these species are significantly influenced by both exogenous and endogenous factors, including age, sex, blood collection site (e.g., tail, eye, heart), pathogen infections, and stressors [1,2,32,33,34], significant discrepancies in immune parameters exist between laboratory strains of mice and rats and their wild counterparts [1,2,35]. For instance, laboratory Balb/c mice infected with murine gammaherpesvirus strain 72 exhibited elevated RL percentages compared to healthy controls, resembling the hematological profile of human infectious mononucleosis [33], a condition characterized by the presence of Downey-type RLs [36].

In wild populations of the genera *Apodemus*, *Microtus* and *Clethrionomys*, numerous studies have investigated the effects of exogenous and endogenous factors on hematological indicators, yielding valuable data on WBC proportions. Seasonality, closely associated with reproductive status, is a factor that significantly alters leukocyte profiles, affecting mainly lymphocyte and neutrophil levels, alongside erythrocyte counts [12,37,38,39]. Age appears to be another critical determinant; younger individuals typically exhibit higher granulocyte and lower lymphocyte counts compared to older ones [37,39,40,41]. In recent years, considerable attention has been directed toward the effects of environmental pollution (e.g., radiation, organic pollutants and heavy metals) on the leukocyte types of these genera. Because they present immediate and pronounced shifts in WBC proportions following exposure, these rodents could potentially serve as effective bioindicators of environmental contamination [41,42,43,44]. Furthermore, intense anthropogenic activity and changes in land use induce acute or chronic stress, resulting in significant fluctuations in leukocyte proportions [28,45,46,47]. Finally, parasitic infections and vector-borne pathogens actively stimulate the immune system, causing notable alterations in hematological parameters [48,49,50].

Collectively, the above-mentioned studies represent only a fraction of the research aimed at determining the precise roles of diverse exogenous and endogenous factors in modulating hematological indices and, consequently, immune responses in wild *Apodemus*, *Microtus* and *Clethrionomys* individuals. This clearly demonstrates that variations in blood cell proportions constitute a highly complex, multifactorial phenomenon. Consequently, determining which factor—or combination of factors—exerts the greatest influence on shaping the definitive hematological profile of wild rodents remains a difficult challenge.

To address these knowledge gaps and provide comparative baseline data, the present study quantified and compared leukocyte proportions among selected wild rodent species belonging to Muridae and Arvicolinae. We formulated two *a priori* hypotheses. First, we hypothesized that leukocyte profiles would differ between the sampled Muridae and Arvicolinae and among species, reflecting taxon-specific physiological and ecological differences. Second, because sex has been reported as a potential determinant of leukocyte variation in rodents, we hypothesized that leukocyte proportions could differ between males and females within each species. In addition to these hypothesis-driven comparisons, we conducted an exploratory morphological assessment of atypical lymphocyte forms to document their occurrence and evaluate whether their distribution appeared restricted to particular taxa. Together, these analyses aim to expand the available baseline hematological information for free-living rodents and provide a foundation for future eco-immunological studies.

## 2. Materials and Methods

### 2.1. Field Sampling

**Figure 1:**
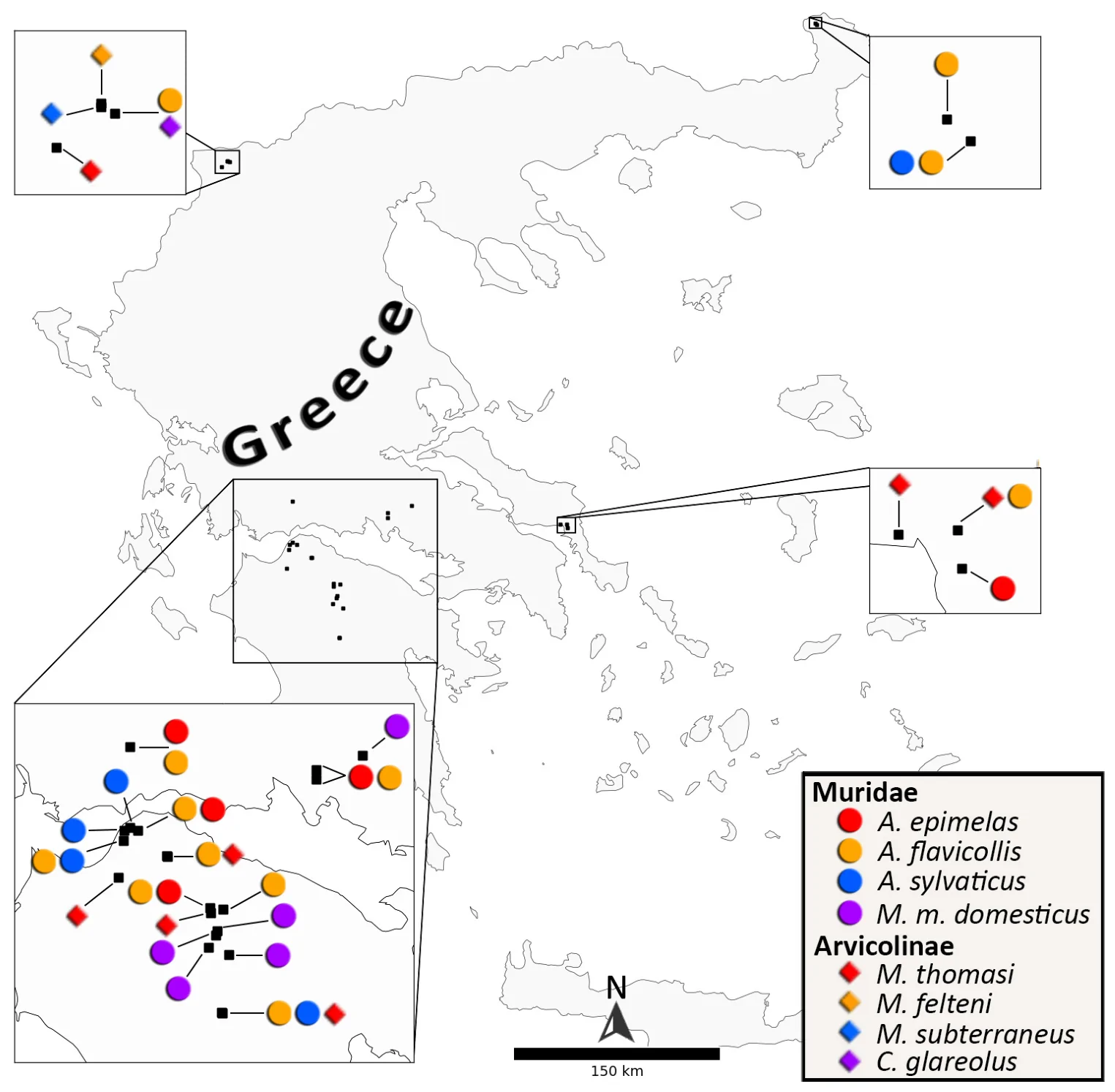
Map of the sampling sites for wild rodents in mainland Greece, indicating the species collected at each sampling location.

A total of 113 wild rodents were captured during the active seasons (May–November) between 2020 and 2024 from locations across seven prefectures of mainland Greece (Figure 1). The animals belonged to the family Muridae (45 *Apodemus flavicollis*, 17 *A. epimelas,* 6 *A. sylvaticus,* 12 *Mus musculus domesticus*) and the subfamily Arvicolinae (27 *Microtus thomasi,* 1 *M. subterraneus,* 1 *M. felteni,* 4 *Clethrionomys glareolus*). Animals were live-trapped, using either Sherman traps (for *M. m. domesticus, Apodemus* spp. and *C. glareolus*) or custom-made traps, specifically designed for the subterranean species of the genus *Microtus.* Detailed information regarding all individuals and their trapping locations is provided in Tables S1 and S2. Following handling and material collection, all wild animals were released at their capture sites.

### 2.2. Blood Collection and Sample Preparation

**Figure 2:**
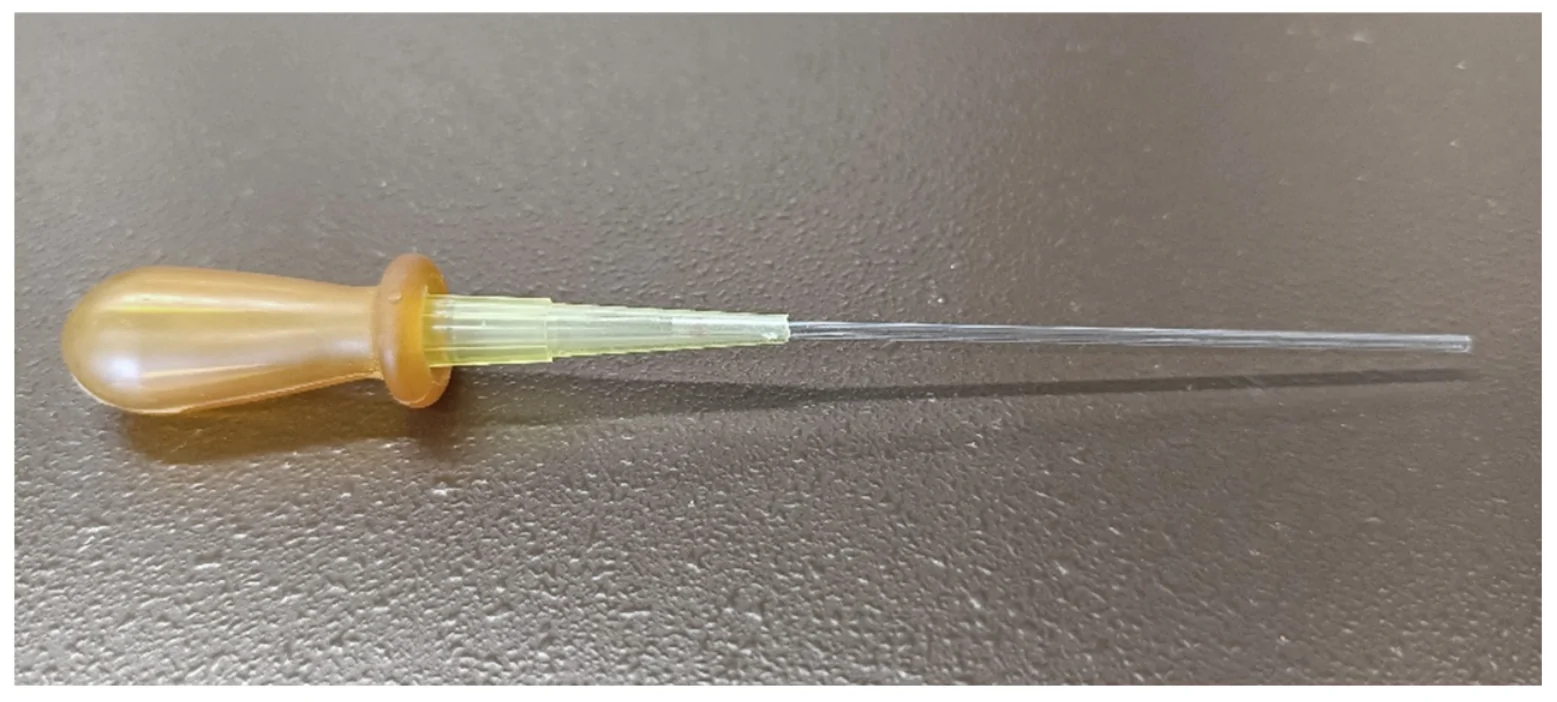
Custom-made tool used during the blood-drawing process.

Animals were anesthetized using ketamine (Ketamidor^®^, VetViva Richter GmbH, Wels, Austria) and medetomidine hydrochloride (Domitor^®^, Orion Corporation, Espoo, Finland) and recovery was facilitated with the administration of atipamezole (Antisedan^®^, Orion Corporation, Espoo, Finland). All three drugs were diluted 1:10 with sterile saline before administration. The administered doses were 50 mg/kg, 1 mg/kg, and 5 mg/kg, respectively, following published protocols [51]. During anesthesia, body measurements, weight and sex were recorded for each specimen (Tables S3 and S4). Prior to measurements, a small quantity of blood was drawn from the saphenous veins of both lower limbs [52], following a minor needle puncture. To achieve this, blood was collected using a heparinized micro-hematocrit capillary tube, mounted on a custom-made tool, consisting of a trimmed 200 μL pipette tip fitted with a 2 mL pipette rubber bulb (Figure 2). This tool was devised to secure rapid and continuous blood flow directly into a Mini EDTA K3 collection tube (MiniCollect^®^ TUBE 0.25/0.5 mL K3E K3EDTA, Greiner Bio-One GmbH, Kremsmünster, Austria).

Two to three blood smears per individual were immediately prepared and fixed by immersion in pure methanol (CH_3_OH; Methanol uniLAB™, Merck/Sigma-Aldrich, Darmstadt, Germany) for one minute. The smears were subsequently stained with Giemsa (LOT: HX46103104, Sigma-Aldrich/Merck, Germany), diluted in a buffer solution at pH 6.8 [0.42 g Gurr Buffer (Gibco™/Thermo Fisher Scientific, Waltham, MA, USA) dissolved in 300 mL dH_2_O]. The dilution ratio was one drop of Giemsa stain per 1 mL of buffer solution. Finally, the smears were mounted using DPX mounting medium (Sigma-Aldrich/Merck, Darmstadt, Germany).

### 2.3. Laboratory Analysis

All blood smears were examined under an optical microscope (Zeiss Axioskop 2 Plus; Carl Zeiss AG, Oberkochen, Germany) equipped with a digital camera (Zeiss AxioCam MRc5; Carl Zeiss AG, Oberkochen, Germany), to evaluate the morphology and proportion of WBCs. For each individual belonging to the family Muridae, two blood smears were examined. In each smear, 100 WBCs were counted, and detailed counts were recorded for lymphocytes, neutrophils, monocytes, eosinophils, basophils, and reactive lymphocytes [53]. The leukocyte proportions obtained from the two smears were averaged for each individual, and the resulting mean values were used in the subsequent statistical analyses. Accordingly, each animal constituted a single independent statistical unit.

For individuals of the subfamily Arvicolinae, two smears were similarly evaluated. However, 200 WBCs per smear were counted, and the resulting leukocyte proportions were subsequently converted to relative proportions (expressed as percentages), to enable direct comparison with data from the Muridae family. This extended cell count was implemented to maximize the probability of detecting AZs, which typically occur at very low frequencies [24,25,30].

### 2.4. Statistical Analysis

Comparative analyses were performed to evaluate variation in leukocyte proportions in relation to taxonomy and sex. Comparisons included: (1) species within the genus *Apodemus*, (2) species within the subfamily Arvicolinae with sufficient sample sizes for inferential analysis, (3) the Muridae family and the Arvicolinae subfamily, and (4) male and female individuals within each species for which adequate sample sizes were available. *Microtus felteni* and *M. subterraneus* were excluded from inferential comparisons because each was represented by a single individual.

Data normality was assessed using the Shapiro–Wilk test. Depending on data distribution and the number of groups compared, either parametric (independent-samples *T*-test) or non-parametric tests (Kruskal–Wallis *H* test or Mann–Whitney *U* test) were applied using IBM SPSS Statistics software (Version 29). Non-parametric tests were used whenever the assumption of normality was not met in at least one of the compared groups. When a Kruskal–Wallis test indicated significant interspecific variation, post-hoc pairwise comparisons were subsequently performed using Mann–Whitney *U* tests.

To account for multiple testing across leukocyte variables, *p*-values were adjusted using the Holm procedure to control the family-wise error rate. Parallel tests of different leukocyte variables addressing the same biological comparison were treated as a single family of hypotheses. Accordingly, Holm adjustment was applied separately to the leukocyte comparisons among *Apodemus* species, between the analyzed Arvicolinae species, between Muridae and Arvicolinae, and to male–female comparisons within each species. For significant Kruskal–Wallis tests, Holm adjustment was additionally applied to the corresponding post-hoc pairwise species comparisons within each leukocyte variable. All tests were two-tailed, and statistical significance was accepted at Holm-adjusted *p* < 0.05. Full raw and Holm-adjusted *p*-values for all statistical comparisons are provided in Table S5.

## 3. Results

The hematological evaluation of the studied wild rodents revealed distinct, lineage-specific patterns in both leukocyte morphology and relative cellular proportions. Across all examined taxa, lymphocytes constituted the predominant leukocyte population, followed by neutrophils. However, significant qualitative and quantitative variations were recorded between the family Muridae and the subfamily Arvicolinae. Notably, peripheral blood smears from *Microtus* species generally appeared to contain fewer white blood cells (WBCs) based on microscopic examination compared to the other studied groups.

### 3.1. Leukocyte Morphology

**Figure 3:**
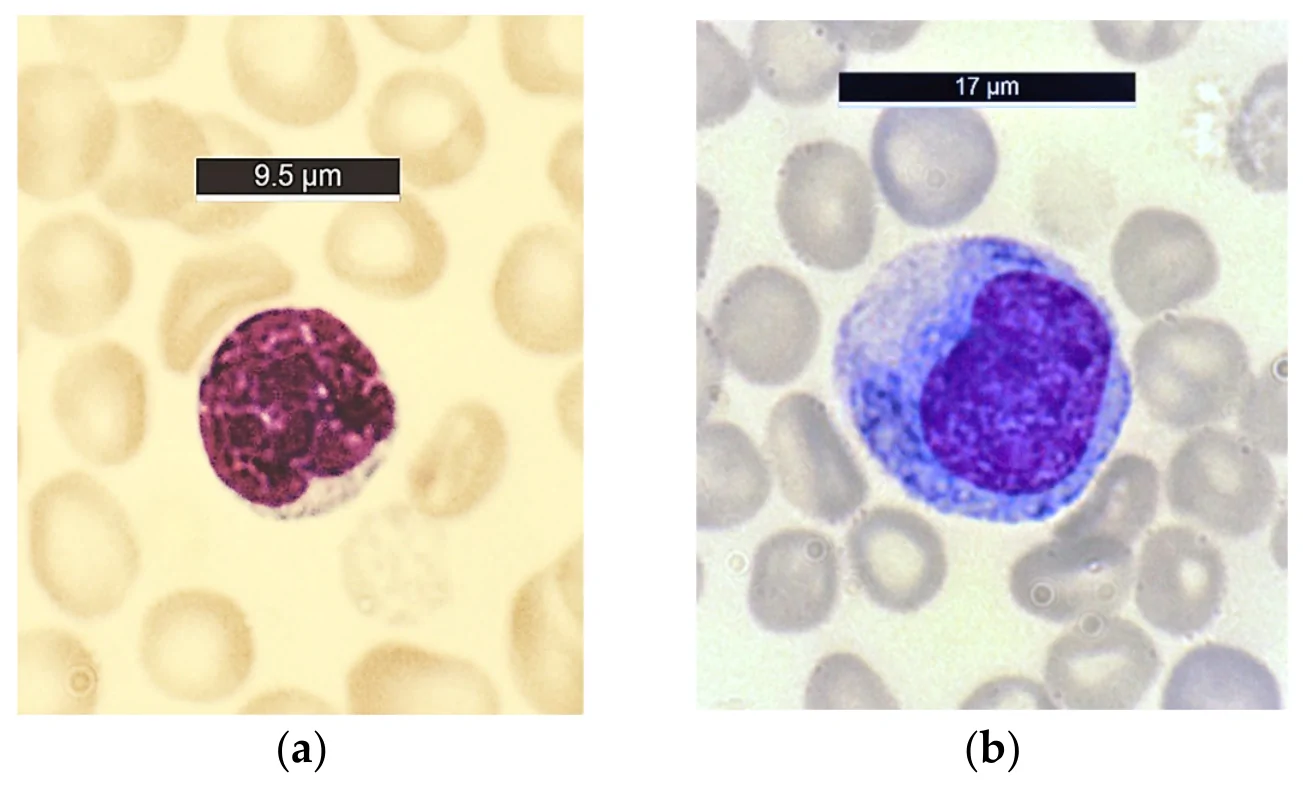
(**a**) A typical lymphocyte and (**b**) a reactive lymphocyte (Downey-type) from blood smears of *M. m. domesticus* individuals.

Regarding leukocyte morphology, wild *M. m. domesticus* individuals presented both typical lymphocytes and RLs of the Downey type. The diameter of these RLs ranged between 15 and 19 μm (Figure 3b), in contrast to typical lymphocytes, which measured approximately 10 μm (Figure 3a). Downey-type cells were also detected at lower percentages in some *Apodemus* individuals but were entirely absent from rodents of the subfamily Arvicolinae.

**Figure 4:**
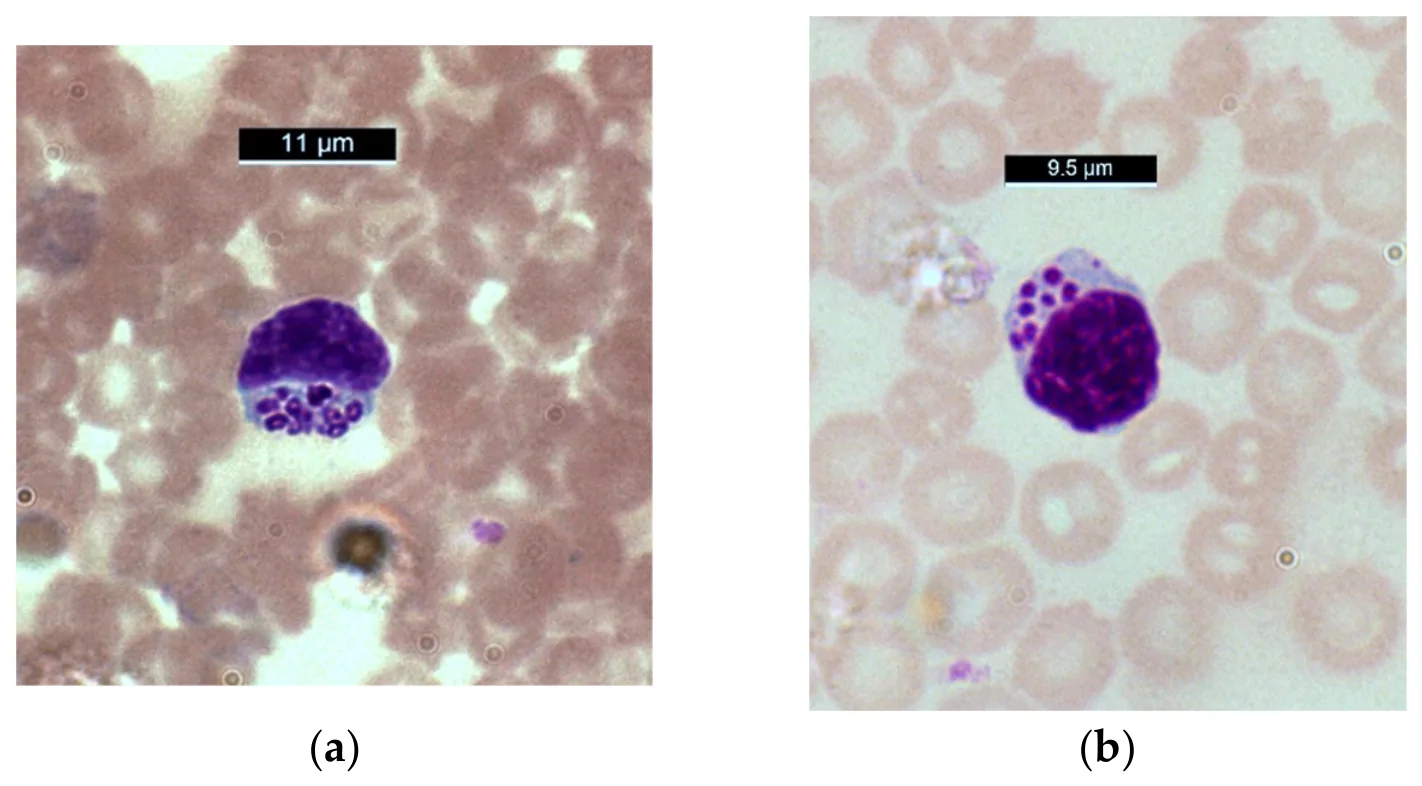
Cells morphologically consistent with azurocytes observed in a peripheral blood smear of a *Microtus thomasi* individual (**a**,**b**).

Cells morphologically consistent with AZs were observed at varying frequencies in 20 adult *Microtus* individuals. Specifically, such cells were observed in blood smears from eleven male and seven female *M. thomasi* individuals, as well as in the single examined male *M. subterraneus* and *M. felteni* individuals. In 18 of these 20 individuals, the proportion of these cells was <1.5%. However, in two adult *M. thomasi* females—both of which were pregnant at the time of capture—the proportions were considerably higher (9.5% and 3.25%, respectively). The observed cells ranged from 9 to 12 μm in diameter and exhibited conspicuous cytoplasmic granules consistent with previously described azurocyte morphology (Figure 4a,b).

Additionally, cells exhibiting a morphological likeness to AZs were observed at very low proportions (<1.5%) in the blood smears of three *C. glareolus* adults.

### 3.2. Proportions of Leukocyte Types

#### 3.2.1. Species of the Genus *Apodemus*

**Figure 5:**
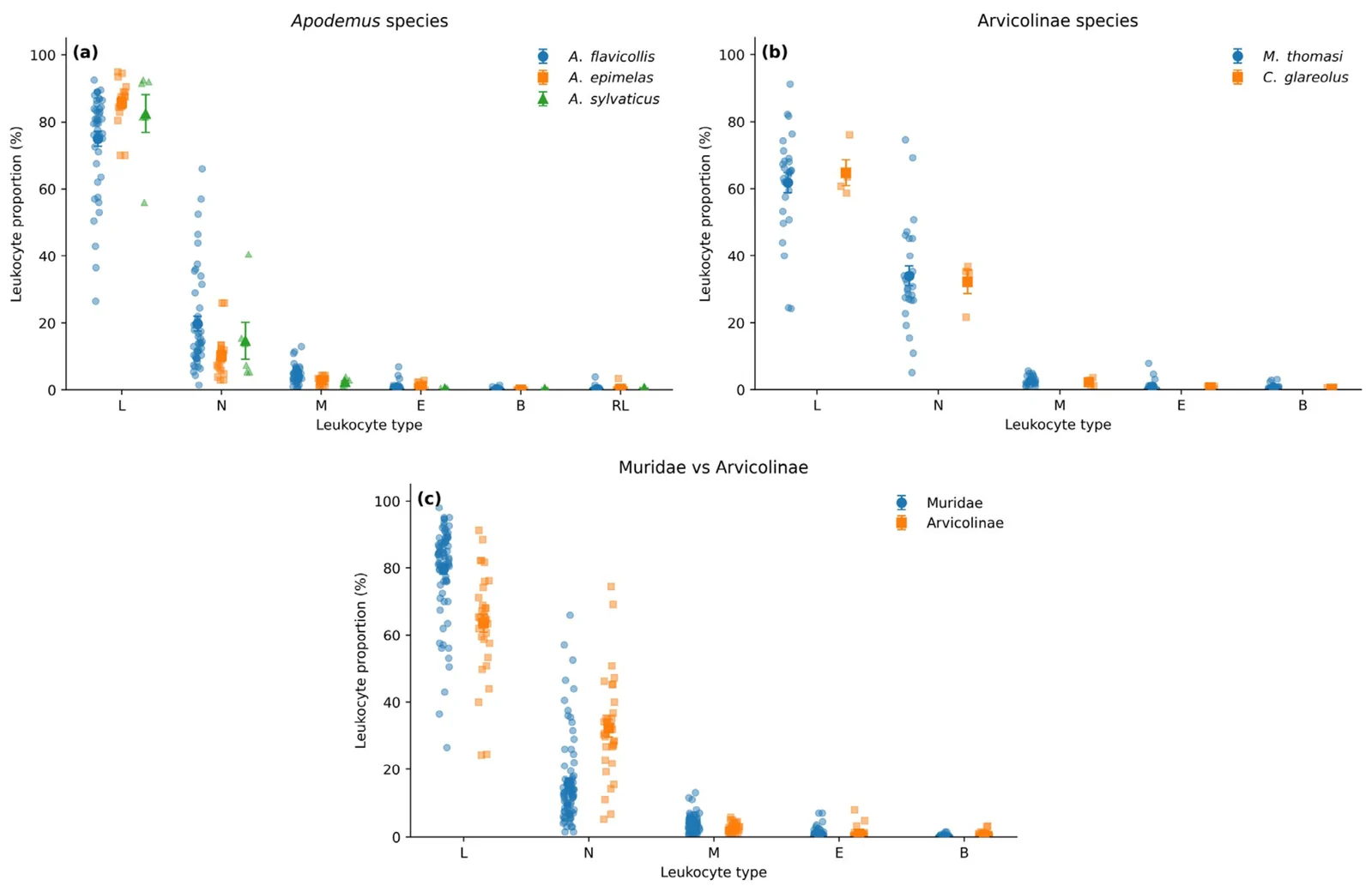
Leukocyte proportions in relation to taxonomy: (**a**) *Apodemus* species, (**b**) Arvicolinae species, and (**c**) Muridae vs. Arvicolinae. Individual observations are shown together with mean ± SEM. L: lymphocyte; N: neutrophil; M: monocyte; E: eosinophil; B: basophil; RL: reactive lymphocyte.

In total, 68 individuals representing the three studied *Apodemus* species (*A. flavicollis*, *A. epimelas* and *A. sylvaticus*) were included in the comparative analysis of leukocyte proportions. The mean ± SEM leukocyte profiles for each species are presented in Figure 5a. After excluding the RL value of one *A. sylvaticus* individual as a statistical outlier (Figure S1) according to Tukey’s 1.5 × IQR criterion, Kruskal–Wallis *H* tests were used to evaluate differences among the three species. Specifically, for RL values, a sensitivity analysis was also performed by repeating the Kruskal–Wallis test with this observation included. The statistical conclusion remained unchanged, with no significant differences among species (with the observation included: *H* = 4.712, *p* = 0.095; after exclusion: *H* = 1.839, *p* = 0.399). After Holm adjustment for multiple comparisons across leukocyte types, significant interspecific differences remained for lymphocytes (*p* = 0.016), neutrophils (*p* = 0.047), monocytes (*p* = 0.047), and eosinophils (*p* = 0.016), whereas basophils and RLs did not differ significantly (*p* = 0.453 for both). Basophils were absent in all *A. epimelas* individuals.

Holm-adjusted post-hoc pairwise comparisons showed that *A. flavicollis* and *A. epimelas* differed significantly in lymphocyte (*p* = 0.003), neutrophil (*p* = 0.010), monocyte (*p* = 0.049), and eosinophil proportions (*p* = 0.005). In addition, *A. epimelas* and *A. sylvaticus* differed significantly in eosinophil proportions (*p* = 0.013). No other pairwise comparison was statistically significant after Holm adjustment. Full raw and Holm-adjusted *p*-values are provided in Table S5.

#### 3.2.2. Species of the Subfamily Arvicolinae

A total of 31 individuals were included in the comparison of leukocyte proportions between *M. thomasi* (*n* = 27) and *C. glareolus* (*n* = 4). *Microtus felteni* and *M. subterraneus* were excluded from inferential analyses, as each was represented by a single individual. The mean ± SEM leukocyte proportions of the two species are presented in Figure 5b. Depending on data distribution, either the Mann–Whitney *U* test or the independent-samples *t*-test were applied to individual leukocyte types. After Holm adjustment for multiple comparisons across leukocyte types, no statistically significant differences were detected between *M. thomasi* and *C. glareolus* (*p* > 0.05; Table S5).

#### 3.2.3. Muridae vs. Arvicolinae Species

A total of 113 individuals were included in the comparison of leukocyte proportions between Muridae (*n* = 80) and Arvicolinae (*n* = 33). The mean ± SEM leukocyte percentages of the two taxonomic groups are presented in Figure 5c. Based on the distribution of the data, Mann–Whitney *U* tests were used to compare leukocyte proportions. After Holm adjustment for multiple comparisons across leukocyte types, significant differences were retained for lymphocytes (*p* < 0.001), neutrophils (*p* < 0.001), and basophils (*p* < 0.001), whereas monocyte and eosinophil proportions did not differ significantly (*p* > 0.05; Table S5).

#### 3.2.4. Comparison Between Male and Female Individuals in Each Species

**Figure 6:**
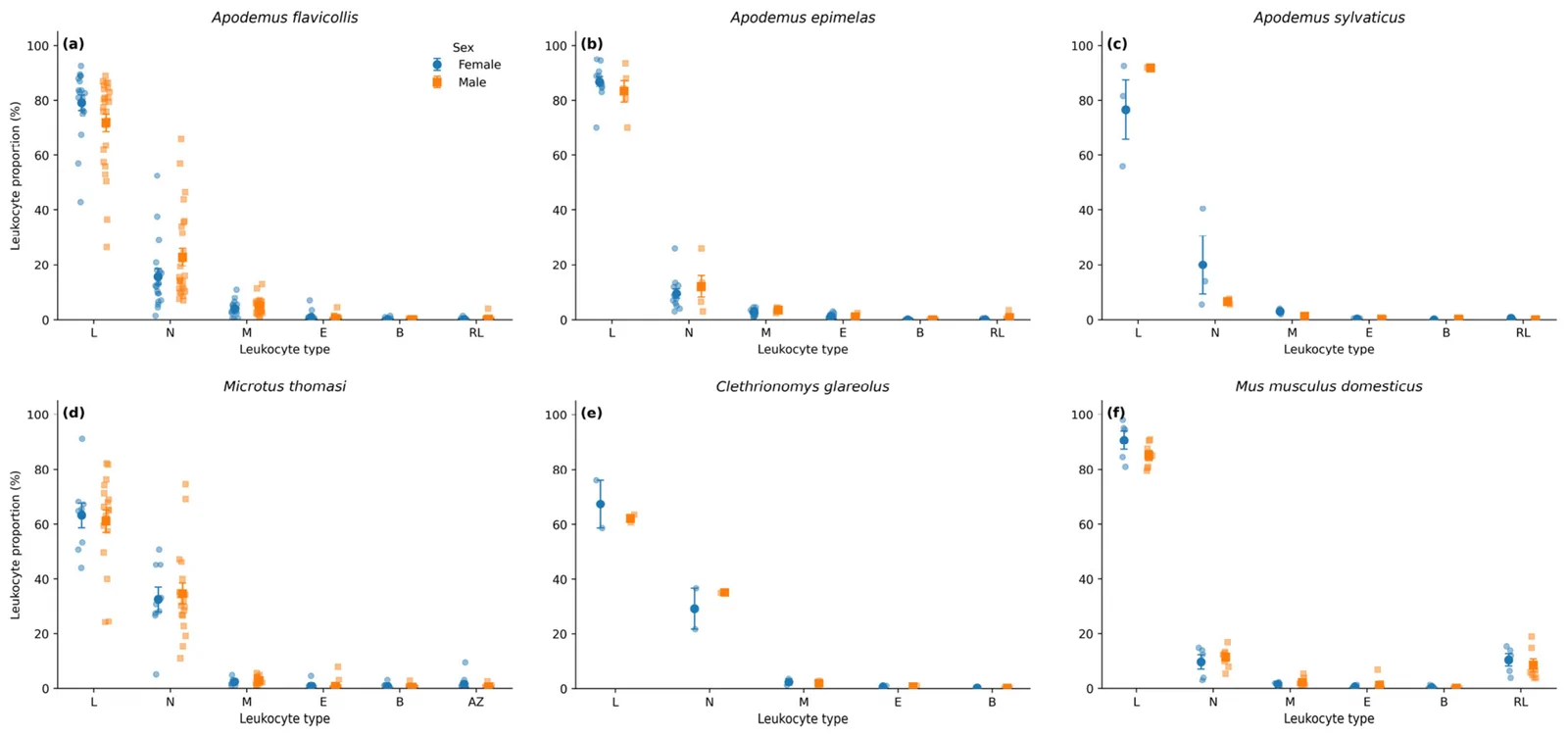
Leukocyte proportions in female and male individuals of the examined species: (**a**) *Apodemus flavicollis*, (**b**) *A. epimelas*, (**c**) *A. sylvaticus*, (**d**) *Microtus thomasi*, (**e**) *Clethrionomys glareolus*, and (**f**) *Mus musculus domesticus*. Individual observations are shown together with mean ± SEM. L: lymphocyte; N: neutrophil; M: monocyte; E: eosinophil; B: basophil; RL: reactive lymphocyte; AZ: cell morphologically consistent with azurocytes.

Potential sex-related variation in leukocyte proportions was evaluated within *A. flavicollis*, *A. epimelas*, *A. sylvaticus*, *M. thomasi*, *C. glareolus*, and *M. m. domesticus*. Again, *M. felteni* and *M. subterraneus* were excluded from these comparisons as each was represented by a single individual. Depending on data distribution, either independent-samples *t*-tests or Mann–Whitney U tests were applied to individual leukocyte types. After Holm adjustment for multiple comparisons within each species, no statistically significant differences in leukocyte proportions were detected between males and females in any of the examined species (*p* > 0.05; Table S5). Mean ± SEM leukocyte profiles according to sex are presented in Figure 6.

## 4. Discussion

The present study provides a comparative hematological assessment of wild rodent species belonging to the family Muridae and the subfamily Arvicolinae. The findings reveal substantial variation in leukocyte morphology and relative proportions among the sampled taxa, while also documenting atypical lymphocyte forms of potential taxonomic interest. Most notably, cells morphologically consistent with AZs were observed in *M. thomasi*, *M. subterraneus*, and *M. felteni,* extending the currently documented occurrence of this leukocyte morphology within the genus *Microtus*. Furthermore, reactive lymphocytes were observed only in Muridae but not in the sampled Arvicolinae specimens. Together, these observations provide new comparative baseline data for wild rodents and highlight the value of species-specific hematological characterization in eco-immunological research.

With regard to the studied wild *M. m. domesticus* and *Apodemus* individuals, the presence of Downey-type RLs, even at low percentages, aligns with their steady exposure to environmental factors that stimulate their immune system [20,54,55]. This observation is consistent with a previous study, suggesting that RL proliferation in peripheral blood acts as a direct indicator of stimulation by infectious agents [33]. Notably, the elevated RL proportions (>10%) were observed in a localized cluster of wild *M. m. domesticus* individuals from Achaia, NW Peloponnese. Although the underlying cause cannot be determined from the present data, such elevated values may indicate recent or ongoing immune stimulation associated with local biological or environmental conditions.

Conversely, RLs were not detected in any of the sampled Arvicolinae individuals, in contrast to their occurrence in Muridae. To the best of our knowledge, the presence of Downey-type cells has not previously been reported in the genera *Microtus* and *Clethrionomys*. This apparent difference is potentially informative, but its biological basis remains unresolved. It may reflect taxon-specific differences in immune activation, ecological exposure, or other physiological factors; however the present study did not aim at distinguishing among these alternatives. Given the limited and uneven sample sizes of the examined Arvicolinae species, except for *Microtus thomasi*, the absence of detected RLs should not be interpreted as evidence that these cells are intrinsically absent from the family. Broader sampling, combined with targeted immunological approaches, will help to determine whether the observed pattern is taxonomically consistent and to clarify its underlying mechanisms.

With regard to AZs, the cells observed in *Microtus* individuals showed morphological characteristics consistent with previous descriptions of AZs, including their relatively large size, the presence of conspicuous cytoplasmic granules, and their characteristic dark-purple appearance following Giemsa staining [24]. Their generally low proportional abundance was also comparable to previously reported patterns in *Microtus* species [24,25,26]. Notably, the considerably higher proportions of these cells in two pregnant *M. thomasi* females fall within ranges previously associated with reproductive status in *M. pennsylvanicus*, where elevated azurocyte proportions have been linked to pregnancy and lactation [56]. Although this observation further supports the morphological interpretation, confirmation of their identity would require targeted histochemical or immunological validation. Subject to this limitation, the observations in *M. thomasi*, *M. subterraneus*, and *M. felteni* appear to extend the currently documented occurrence of cells morphologically consistent with AZs within the genus *Microtus*, beyond the species in which they had been previously reported, i.e., *M. pennsylvanicus* [24], *M. townsendii* [24], *M. oeconomus* [24,27], *M. agrestis* [25], *M. arvalis* [28], and *M. rossiaemeridionalis* [28].

This taxonomic expansion raises compelling questions about the evolutionary origins and true prevalence of AZs. Further investigation is necessary to determine whether this cell type is strictly confined to the genus *Microtus* or whether it extends to other members of the subfamily Arvicolinae. Expanding this screening, starting with phylogenetically closely related taxa—such as the genus *Chionomys*, which is also distributed in Greece (represented by *Ch. syriacus*)—would be particularly valuable. In this context, the recorded cells at very low proportions (<1.5%) in *C. glareolus* with a granular lymphocyte-like morphology differed from the cells morphologically consistent with AZs observed in *Microtus*, particularly in the smaller size of their cytoplasmic granules. Morphologically similar unidentified cells have been previously reported in this species [29], and their appearance may be compatible with LGLs; however, their definitive classification cannot be established from Giemsa-stained smears alone and would also require histochemical and immunological validation. No cells resembling AZs were observed in the sampled Muridae individuals. Although this pattern is consistent with the currently documented distribution of AZs, broader taxonomic sampling will be necessary before firm conclusions can be drawn regarding their restriction to particular rodent lineages.

Consistent with previous related studies [15,31,39,53,57], lymphocytes, followed by neutrophils, constituted the predominant leukocyte types across all studied species. In the present dataset, the significant differences found between the sampled Muridae and Arvicolinae should be interpreted with caution. The two taxonomic groups comprised different species that were represented by unequal sample sizes and were sampled across heterogeneous localities and periods; consequently, the observed patterns cannot be attributed unambiguously to taxonomy alone. Rather, they describe differences between the Muridae and Arvicolinae assemblages represented in the present dataset and may reflect a combination of species composition, ecological characteristics, physiological condition, and other unmeasured environmental or biological factors. Similar variation in leukocyte profiles has been reported among wild rodent taxa [10,28,40,45,47], but broader inference regarding lineage-specific immune strategies will require more-balanced multispecies sampling and designs capable of separating taxonomic effects from ecological and sampling-related variation.

Interestingly, the findings of this work suggest that the baseline leukocyte profile of a species remains notably stable, even under quite different environmental conditions. When significant deviations in primary leukocyte populations do occur, they appear to be direct indicators of acute physiological eustress or pathological distress. For example, a subset of *A. flavicollis* and *M. thomasi* individuals exhibiting an abnormal clinical appearance at capture (lethargy, diminished reflexes), demonstrated marked shifts in lymphocyte and neutrophil proportions. This observation agrees with previous studies, suggesting that these two cell lineages are the most sensitive ones and respond rapidly to exogenous or endogenous stressors, such as acute infections [12,39,40,41,47], whereas other leukocyte types remain largely unaffected.

While baseline leukocyte profiles remained homogeneous between the studied Arvicolinae species, namely *M. thomasi* and *C. glareolus*, variability was found among the studied species of the genus *Apodemus*. The significant differences observed between *A. flavicollis* and *A. epimelas*, as well as between *A. sylvaticus* and *A. epimelas*, suggest that leukocyte profiles may vary substantially even among closely related species. Such differences could reflect species-specific physiological or ecological characteristics, as well as variation in exposure to environmental or biological stressors, although the present sampling design does not allow these potential mechanisms to be disentangled. Interspecific variation in leukocyte profiles within *Apodemus* has also been reported previously, including differences between *A. flavicollis* and *A. agrarius* [42].

It should also be noted that *A. flavicollis* and *A. sylvaticus* are considered ideal bioindicators for studying pollution levels in an area, as their widespread populations, broad habitat utilization, and rapid physiological responsiveness to adverse environmental conditions or to pollution make their dynamic leukocyte profiles a very sensitive indicator of habitat quality [41,58,59,60]. In the present study, however, the small sample size of *A. sylvaticus* limits the strength of species-specific inference for this taxon. Accordingly, the observed leukocyte patterns in *A. sylvaticus* should be regarded as preliminary and require confirmation using larger and more-balanced samples.

Regarding potential sex-related variation, no statistically significant differences in leukocyte proportions were detected between males and females within any of the examined species. Previous studies have reported inconsistent sex-related patterns in rodent leukocyte profiles, with some detecting differences, particularly in lymphocyte proportions [44,47,50], whereas others found no statistically significant sex-related variation [13,38,39,41,61]. Our results are therefore consistent with the latter pattern but should not be interpreted as evidence that sex has no biological influence on leukocyte profiles, keeping in mind the limited and uneven sex-specific sample sizes in several of the studied species.

Despite the baseline parameters established in this study, certain limitations regarding our sampling design and analytical methods should be considered. Regarding sample structure, species representations were uneven, reflecting the inherent challenges in sampling free-living populations with varying natural abundance and trap-shyness. This disparity is particularly pronounced in elusive subterranean mammals, many of which inherently yield low capture rates despite extensive trapping efforts. Thus, *A. sylvaticus* and *C. glareolus* were limited in numbers, while *M. felteni* and *M. subterraneus* were each represented by a single individual and were thus excluded from inferential statistics. This imbalance limits species-level precision and means that higher-level comparisons (such as between Muridae and Arvicolinae) reflect our specific species assemblages rather than definitive family-level traits. Furthermore, sampling was spatially and temporally heterogeneous across localities, habitats, and years, which prevented us from isolating the independent effects of these ecological factors on leukocyte variation. From a physiological perspective, precise age, reproductive status, and health status could not be determined in wild-caught individuals. Because subclinical infections or demographic differences can modulate leukocyte proportions, these unmeasured variables represent potential sources of inter-individual variability. Methodologically, while Giemsa-stained blood smears are reliable for standard morphological profiling, definitive characterization of specialized cell types—such as LGL-like cells in *C. glareolus* and cells morphologically consistent with AZs in *Microtus* species—would require histochemical or immunological marker validation. Finally, differences in the total number of leukocytes counted per smear between Muridae and Arvicolinae may have resulted in minor variations in the precision of estimated proportions. Addressing these methodological challenges through more-standardized, multi-season sampling—while expanding screening to additional taxa within the subfamily Arvicolinae—will be essential to validate these baseline parameters. Such efforts will undoubtedly deepen our understanding of the eco-immunological and evolutionary adaptations of wild rodents.

## 5. Conclusions

This study analyzed blood cell profiles in wild rodent species from Greece, specifically within the family Muridae and the subfamily Arvicolinae. The results show clear differences in leukocyte proportions between these two groups. While lymphocytes were the most common white blood cell type in all species, Arvicolinae exhibited higher neutrophil and lower lymphocyte proportions compared to Muridae.

While leukocyte profiles remained relatively stable within the Arvicolinae group, more variability was observed among *Apodemus* species. No statistically significant differences in leukocyte proportions were detected between males and females within the examined species. A notable finding was the observation of cells morphologically consistent with AZs in *M. thomasi* and in the single examined individuals of *M. felteni* and *M. subterraneus*, although confirmation of their identity will require targeted histochemical or immunological approaches.

These data provide a necessary reference point for hematological studies in wild rodents. By documenting these species-specific immune profiles, this work contributes to the understanding of how wild populations naturally manage their immune responses, supporting the use of these rodents as reliable indicators for environmental and health monitoring.

## Data Availability

The data presented in this study are available in the submitted Supplementary Materials (Tables S1–S5 and Figure S1).

## Supplementary Materials

The following supporting information can be downloaded at: https://www.mdpi.com/article/10.3390/biology15171528/s1; Table S1: Mean values of leukocyte types for each individual of the family Muridae. WBC: white blood cell, L: lymphocyte, N: neutrophil, M: monocyte, E: eosinophil, B: basophil, RL: reactive lymphocyte; Table S2: Mean values of leukocyte types for each individual of the subfamily Arvicolinae. WBC: white blood cell, L: lymphocyte, N: neutrophil, M: monocyte, E: eosinophil, B: basophil, AZ: azurocyte; Table S3: Body measurements of each studied individual of the family Muridae. WBL: whole body length (mm), TL: tail length (mm), EL: ear length (mm), HF: hind foot length (mm), W: weight (g); Table S4: Body measurements of each studied individual of the subfamily Arvicolinae. WBL: whole body length (mm), TL: tail length (mm), EL: ear length (mm), HF: hind foot length (mm), W: weight (g); Table S5: Results of statistical comparisons of leukocyte proportions, including raw and Holm-adjusted *p*-values, and Figure S1: Box plot diagram of the mean values of reactive lymphocytes of the genus *Apodemus*.
