# Supplementary material for: Comparative Analysis of Leukocyte Proportions and Morphology in Wild Muridae and Arvicolinae Rodents
Source: Biology (Basel). 2026 Sep 3;15(17):1528. doi: 10.3390/biology15171528 (PMC13564663; doi:10.3390/biology15171528)
Supplement: Supplementary file 1 [file biology-15-01528-s001.zip › biology-4502569-supplementary.pdf]

SUPPLEMENTARY DATA

Table S1. Mean values of leukocyte types for each individual of the family Muridae. WBC: White blood cell, L: Lymphocyte, N: Neutrophil, M: Monocyte, E: Eosinophil, B: Basophil, RL: Reactive Lymphocyte.

No.	Species	Sex	Collection Date	Locality	Coordinates		Mean values of leukocyte types (%)						
					Longitude	Latitude	WBC	L	N	M	E	B	RL
1	*A. flavicollis*	♀	21/9/2020	Aroi, Patras	21.764	38.242	100	89.0	6.5	0.5	3.5	0.5	0.0
2	*A. flavicollis*	♂	20/10/2020	Ano Kastritsi	21.826	38.275	100	89.0	8.5	2.5	0.0	0.0	0.0
3	*A. flavicollis*	♀	30/5/2021	Aroi, Patras	21.764	38.242	100	67.5	29.0	2.5	1.0	0.0	0.0
4	*A. flavicollis*	♀	30/5/2021	Aroi, Patras	21.764	38.242	100	89.0	10.5	0.5	0.0	0.0	0.0
5	*A. flavicollis*	♀	31/5/2021	Aroi, Patras	21.764	38.242	100	92.5	4.5	2.5	0.5	0.0	0.0
6	*A. flavicollis*	♀	16/7/2021	Kalavryta ski resort	22.189	38.012	100	83.5	12.0	4.0	0.5	0.0	0.5
7	*A. flavicollis*	♀	17/7/2021	Aesthetic Forest of Kalavryta	22.134	38.018	100	87.5	8.5	3.5	0.5	0.0	0.0
8	*A. flavicollis*	♀	16/7/2021	Kalavryta ski resort	22.189	38.012	100	87.0	9.5	3.5	0.0	0.0	0.0
9	*A. flavicollis*	♂	16/7/2021	Kalavryta ski resort	22.189	38.012	100	75.0	21.0	4.0	0.0	0.0	0.0
10	*A. flavicollis*	♂	17/7/2021	Aesthetic Forest of Kalavryta	22.136	38.018	100	77.5	16.0	5.0	1.5	0.0	0.0
11	*A. flavicollis*	♂	17/7/2021	Aesthetic Forest of Kalavryta	22.134	38.018	100	84.5	14.0	1.5	0.0	0.0	0.0
12	*A. flavicollis*	♂	14/9/2021	Aroi, Patras	21.764	38.242	100	50.5	46.5	3.0	0.0	0.0	0.5
13	*A. flavicollis*	♂	14/9/2021	Aroi, Patras	21.764	38.242	100	87.0	12.0	1.0	0.0	0.0	0.0
14	*A. flavicollis*	♂	16/9/2021	Aroi, Patras	21.764	38.242	100	76.0	19.5	3.5	1.0	0.0	0.0
15	*A. flavicollis*	♂	16/9/2021	Aroi, Patras	21.764	38.242	100	81.0	15.5	3.5	0.0	0.0	0.0
16	*A. flavicollis*	♂	4/10/2021	Arachova	22.587	38.479	100	79.5	10.0	6.0	4.5	0.0	0.0
17	*A. flavicollis*	♀	5/10/2021	Arachova	22.576	38.487	100	82.5	5.5	11.0	1.0	0.0	0.0
18	*A. flavicollis*	♀	6/10/2021	Arachova	22.598	38.478	100	84.0	14.0	1.0	1.0	0.0	0.5
19	*A. flavicollis*	♂	6/10/2021	Arachova	22.598	38.478	100	36.5	57.0	6.5	0.0	0.0	0.0
20	*A. flavicollis*	♂	29/10/2021	Dorvitsa	21.793	38.555	100	79.5	7.0	13.0	0.0	0.5	0.0
21	*A. flavicollis*	♂	12/4/2022	Krini, Mt. Panachaiko	21.951	38.189	100	53.0	44.0	3.0	0.0	0.0	0.5
22	*A. flavicollis*	♂	12/4/2022	Krini, Mt. Panachaiko	21.951	38.189	100	83.0	14.5	2.0	0.5	0.0	0.0
23	*A. flavicollis*	♀	12/4/2022	Krini, Mt. Panachaiko	21.951	38.189	100	76.0	17.0	5.5	0.5	1.0	0.0
24	*A. flavicollis*	♂	12/4/2022	Krini, Mt. Panachaiko	21.951	38.189	100	26.5	66.0	7.0	0.0	0.5	0.0
25	*A. flavicollis*	♂	12/4/2022	Krini, Mt. Panachaiko	21.951	38.189	100	56.0	36.0	6.5	1.0	0.5	0.0

No.	Species	Sex	Collection Date	Locality	Coordinates		Mean values of leukocyte types (%)						
					Longitude	Latitude	WBC	L	N	M	E	B	RL
26	*A. flavicollis*	♀	12/4/2022	Krini, Mt. Panachaiko	21.951	38.189	100	43.0	52.5	3.0	0.0	1.5	0.0
27	*A. flavicollis*	♂	29/5/2022	Vytina	22.185	37.665	100	57.5	35.5	7.0	0.0	0.0	0.0
28	*A. flavicollis*	♂	29/5/2022	Vytina	22.185	37.665	100	71.0	22.0	5.5	1.5	0.0	0.0
29	*A. flavicollis*	♂	29/5/2022	Vytina	22.185	37.665	100	86.5	10.5	2.5	0.5	0.0	0.0
30	*A. flavicollis*	♂	20/4/2023	Pentalofos	26.159	41.672	100	85.5	9.5	5.0	0.0	0.0	0.0
31	*A. flavicollis*	♂	20/4/2023	Petroto	26.145	41.682	100	81.0	14.0	4.5	0.5	0.0	0.0
32	*A. flavicollis*	♂	10/5/2023	Aliveri	24.077	38.407	100	62.0	31.5	6.5	0.0	0.0	0.0
33	*A. flavicollis*	♀	9/5/2023	Aliveri	24.077	38.407	100	88.0	7.0	5.0	0.0	0.0	0.0
34	*A. flavicollis*	♀	8/5/2023	Aliveri	24.077	38.407	100	80.5	9.5	3.0	7.0	0.0	0.0
35	*A. flavicollis*	♂	10/5/2023	Aliveri	24.077	38.407	100	81.0	12.5	6.5	0.0	0.0	0.0
36	*A. flavicollis*	♂	16/7/2023	Vigla ski resort	21.267	40.774	100	86.5	7.5	5.5	0.0	0.5	4.0
37	*A. flavicollis*	♀	16/7/2023	Vigla ski resort	21.267	40.774	100	83.0	13.0	3.5	0.5	0.0	0.0
38	*A. flavicollis*	♀	16/7/2023	Vigla ski resort	21.267	40.774	100	57.0	37.5	4.5	1.0	0.0	0.0
39	*A. flavicollis*	♂	16/7/2023	Vigla ski resort	21.267	40.774	100	72.5	24.5	3.0	0.0	0.0	0.5
40	*A. flavicollis*	♂	16/7/2023	Vigla ski resort	21.267	40.774	100	84.0	11.5	4.5	0.0	0.0	0.0
41	*A. flavicollis*	♀	16/7/2023	Vigla ski resort	21.267	40.774	100	76.5	18.0	5.5	0.0	0.0	0.0
42	*A. flavicollis*	♂	16/7/2023	Vigla ski resort	21.267	40.774	100	63.5	34.0	2.5	0.0	0.0	0.0
43	*A. flavicollis*	♂	16/7/2023	Vigla ski resort	21.267	40.774	100	76.0	11.5	11.5	1.0	0.0	0.0
44	*A. flavicollis*	♀	16/7/2023	Vigla ski resort	21.267	40.774	100	79.5	17.5	3.0	0.0	0.0	0.0
45	*A. flavicollis*	♀	16/7/2023	Vigla ski resort	21.267	40.774	100	89.5	1.5	8.0	1.0	0.0	1.5
46	*A. epimelas*	♂	21/10/2020	Ano Kastritsi	21.826	38.275	100	88.0	6.5	4.5	1.0	0.0	3.5
47	*A. epimelas*	♀	19/10/2020	Ano Kastritsi	21.826	38.275	100	86.0	9.0	4.5	0.5	0.0	0.0
48	*A. epimelas*	♀	19/10/2020	Ano Kastritsi	21.826	38.275	100	89.0	7.5	3.5	0.0	0.0	0.5
49	*A. epimelas*	♀	17/7/2021	Aesthetic Forest of Kalavryta	22.134	38.018	100	94.5	4.0	1.0	0.5	0.0	0.0
50	*A. epimelas*	♂	17/7/2021	Aesthetic Forest of Kalavryta	22.134	38.018	100	94.5	4.0	1.0	0.5	0.0	0.0
51	*A. epimelas*	♀	17/7/2021	Aesthetic Forest of Kalavryta	22.136	38.018	100	93.5	3.0	2.5	1.0	0.0	0.5
52	*A. epimelas*	♀	1/8/2021	Arachova	22.586	38.448	100	80.5	14.0	5.5	0.0	0.0	0.0
53	*A. epimelas*	♀	2/9/2021	Arachova	22.586	38.448	100	85.0	13.5	1.0	0.5	0.0	0.0
54	*A. epimelas*	♀	6/10/2021	Arachova	22.598	38.478	100	89.0	5.0	4.5	1.5	0.0	0.0
55	*A. epimelas*	♀	6/10/2021	Arachova	22.598	38,478	100	87.5	7.0	3.5	2.0	0.0	0.0
56	*A. epimelas*	♂	31/10/2021	Dorvitsa	21.793	38.555	100	80.5	13.5	3.5	2.5	0.0	0.0

No.	Species	Sex	Collection Date	Locality	Coordinates		Mean values of leukocyte types (%)						
					Longitude	Latitude	WBC	L	N	M	E	B	RL
57	*A. epimelas*	♂	1/11/2021	Dorvitsa	21.788	38.555	100	70.0	26.0	3.5	0.5	0.0	0.0
58	*A. epimelas*	♂	9/5/2023	Aliveri	24.081	38.381	100	84.5	11.5	3.5	0.5	0.0	0.0
59	*A. epimelas*	♂	8/5/2023	Aliveri	24.081	38.381	100	84.5	12.0	2.5	1.0	0.0	0.0
60	*A. epimelas*	♀	9/5/2023	Aliveri	24.081	38.380	100	90.5	6.0	2.5	1.0	0.0	0.0
61	*A. epimelas*	♀	9/5/2023	Aliveri	24.081	38.380	100	70.0	26.0	1.5	2.5	0.0	0.0
62	*A. epimelas*	♀	8/5/2023	Aliveri	24.081	38.380	100	83.0	12.5	1.5	3.0	0.0	0.0
63	*A. sylvaticus*	♂	10/10/2020	University of Patras Campus	21.792	38.286	100	91.5	7.5	1.0	0.0	0.0	0.0
64	*A. sylvaticus*	♀	10/10/2020	University of Patras Campus	21.792	38.286	100	81.5	14.0	4.0	0.5	0.0	1.0
65	*A. sylvaticus*	♀	12/10/2020	Aroi, Patras	21.764	38.242	100	92.5	5.5	2.0	0.0	0.0	0.5
66	*A. sylvaticus*	♂	16/12/2020	Proastio, Patras	21.769	38.275	100	81.5	15.5	3.0	0.0	0.0	61.0
67	*A. sylvaticus*	♀	29/5/2022	Vytina	22.185	37.665	100	56.0	40.5	3.0	0.5	0.0	0.0
68	*A. sylvaticus*	♂	20/4/2023	Pentalofos	26.159	41.672	100	92.0	5.5	1.5	0.5	0.5	0.0
69	*M. m. domesticus*	♂	17/10/2020	Planitero	22.165	37.94	100	81.0	10.0	2.0	7.0	0.0	6.0
70	*M. m. domesticus*	♀	17/10/2020	Lykouria	22.213	37.859	100	94.5	4.0	1.0	0.5	0.0	12.0
71	*M. m. domesticus*	♂	18/10/2020	Kleitoria	22.128	37.885	100	87.5	12.5	0.0	0.0	0.0	19.0
72	*M. m. domesticus*	♂	18/10/2020	Kalamaki	22.157	37.925	100	90.5	5.5	4.0	0.0	0.0	15.0
73	*M. m. domesticus*	♀	18/10/2020	Lykouria	22.213	37.859	100	95.0	3.0	2.0	0.0	0.0	14.0
74	*M. m. domesticus*	♀	18/10/2020	Kalamaki	22.157	37.925	100	98.0	1.5	0.5	0.0	0.0	15.5
75	*M. m. domesticus*	♂	8/10/2021	Mavroneri	22.783	38.526	100	85.0	13.0	2.0	0.0	0.0	4.0
76	*M. m. domesticus*	♀	8/10/2021	Mavroneri	22.783	38.526	100	84.5	12.5	2.5	0.5	0.0	4.0
77	*M. m. domesticus*	♂	8/10/2021	Mavroneri	22.783	38.526	100	79.5	17.0	1.5	2.0	0.0	5.0

No.	Species	Sex	Collection Date	Locality	Coordinates		Mean values of leukocyte types (%)						
					Longitude	Latitude	WBC	L	N	M	E	B	RL
78	*M. m. domesticus*	♂	8/10/2021	Mavroneri	22.783	38.526	100	91.0	8.0	1.0	0.0	0.0	6.5
79	*M. m. domesticus*	♀	8/10/2021	Mavroneri	22.783	38.526	100	81.0	14.0	2.0	1.5	1.5	6.5
80	*M. m. domesticus*	♂	8/10/2021	Mavroneri	22.783	38.526	100	80.5	13.5	5.5	0.0	0.5	4.0

Table S2. Mean values of leukocyte types for each individual of the subfamily Arvicolinae. WBC: White blood cell, L: Lymphocyte, N: Neutrophil, M: Monocyte, E: Eosinophil, B: Basophil, AZ: Azurocyte.

No.	Species	Sex	Collection Date	Locality	Coordinates		Mean values of leukocyte types (%)						
					Longitude	Latitude	WBC	L	N	M	E	B	AZ
1	*M. thomasi*	♀	15/7/2021	Ano Lousoi	22.136	38.000	100	64.00	33.00	2.00	0.50	0.50	1.00
2	*M. thomasi*	♂	16/11/2021	Kydonies, Vasiliko	21.743	38.118	100	65.25	32.00	2.25	0.00	0.50	1.25
3	*M. thomasi*	♂	16/11/2021	Kydonies, Vasiliko	21.743	38.118	100	59.50	35.25	4.00	0.50	0.75	0.50
4	*M. thomasi*	♀	16/11/2021	Kydonies, Vasiliko	21.743	38.118	100	44.00	50.75	5.00	0.25	0.00	9.50
5	*M. thomasi*	♂	16/11/2021	Kydonies, Vasiliko	21.743	38.118	100	76.25	19.25	4.25	0.25	0.00	0.75
6	*M. thomasi*	♂	17/11/2021	Kydonies, Vasiliko	21.743	38.118	100	65.00	29.75	5.00	0.25	0.00	2.75
7	*M. thomasi*	♂	17/11/2021	Kydonies, Vasiliko	21.743	38.118	100	68.00	27.00	2.00	0.00	3.00	0.00
8	*M. thomasi*	♂	17/11/2021	Kydonies, Vasiliko	21.743	38.118	100	49.75	46.25	2.00	1.25	0.75	1.00
9	*M. thomasi*	♀	17/11/2021	Kydonies, Vasiliko	21.743	38.118	100	65.50	28.25	3.00	0.00	3.25	0.25
10	*M. thomasi*	♂	17/11/2021	Kydonies, Vasiliko	21.743	38.118	100	24.50	69.25	5.75	0.00	0.50	0.00
11	*M. thomasi*	♂	11/4/2022	Krini, Mt. Panachaiko	21.950	38.190	100	62.00	34.00	4.00	0.00	0.00	1.00
12	*M. thomasi*	♂	12/4/2022	Krini, Mt. Panachaiko	21.950	38.190	100	24.25	74.50	1.00	0.00	0.25	0.00
13	*M. thomasi*	♂	12/4/2022	Krini, Mt. Panachaiko	21.950	38.190	100	66.25	30.25	3.25	0.25	0.00	0.00
14	*M. thomasi*	♂	12/4/2022	Krini, Mt. Panachaiko	21.950	38.190	100	40.00	47.25	4.50	8.00	0.25	0.00
15	*M. thomasi*	♂	12/4/2022	Krini, Mt. Panachaiko	21.950	38.190	100	71.25	26.75	2.00	0.00	0.00	0.00
16	*M. thomasi*	♂	12/4/2022	Krini, Mt. Panachaiko	21.950	38.190	100	74.25	22.75	2.75	0.00	0.25	1.00
17	*M. thomasi*	♂	12/4/2022	Krini, Mt. Panachaiko	21.950	38.190	100	63.00	34.25	2.25	0.25	0.25	0.50
18	*M. thomasi*	♂	28/5/2022	Vytina	22.184	37.666	100	81.75	15.50	1.50	1.00	0.25	0.25
19	*M. thomasi*	♀	28/5/2022	Vytina	22.184	37.666	100	64.75	26.75	2.50	4.75	1.25	3.25
20	*M. thomasi*	♀	28/5/2022	Vytina	22.184	37.666	100	50.75	45.25	2.75	1.25	0.00	0.75
21	*M. thomasi*	♂	28/5/2022	Vytina	22.184	37.666	100	82.25	11.00	3.00	3.25	0.50	0.25
22	*M. thomasi*	♂	28/5/2022	Vytina	22.184	37.666	100	57.50	40.00	2.50	0.00	0.00	0.50
23	*M. thomasi*	♀	10/5/2023	Aliveri	24.077	38.407	100	91.25	5.25	1.75	0.75	1.00	0.25
24	*M. thomasi*	♀	10/5/2023	Aliveri	24.025	38.404	100	67.25	30.75	1.00	0.50	0.50	0.00
25	*M. thomasi*	♀	10/5/2023	Aliveri	24.025	38.404	100	53.25	45.25	1.00	0.50	0.00	0.00
26	*M. thomasi*	♂	10/5/2023	Aliveri	24.025	38.404	100	69.00	28.50	1.25	1.25	0.00	0.00
27	*M. thomasi*	♀	18/9/2023	Trigono	21.199	40.743	100	68.25	27.50	3.25	0.75	0.25	0.50
28	*C. glareolus*	♂	16/7/2023	Vigla ski resort	21.267	40.774	100	63.50	35.00	1.00	0.25	0.25	1.50

No.	Species	Sex	Collection Date	Locality	Coordinates		Mean values of leukocyte types (%)						
					Longitude	Latitude	WBC	L	N	M	E	B	AZ
29	*C. glareolus*	♀	16/7/2023	Vigla ski resort	21.267	40.774	100	76.00	21.75	1.25	1.00	0.00	0.50
30	*C. glareolus*	♀	16/7/2023	Vigla ski resort	21.267	40.774	100	58.75	36.75	3.75	0.25	0.50	1.25
31	*C. glareolus*	♂	16/7/2023	Vigla ski resort	21.267	40.774	100	60.75	35.25	3.00	1.00	0.00	0.00
32	*M. felteni*	♂	16/7/2023	Pisoderi	21.251	40.783	100	88.50	6.75	2.25	1.00	1.50	1.50
33	*M. subterraneus*	♂	20/7/2023	Pisoderi	21.251	40.780	100	82.25	14.25	2.75	0.25	0.50	2.75

Table S3. Body measurements of each studied individual of the family Muridae. WBL: Whole body length (mm), TL: Tail length (mm), EL: Ear length (mm), HF: Hind foot length (mm), W: Weight (g).

No.	Species	Sex	Collection Date	Locality	Coordinates		Body Measurements				
					Longitude	Latitude	WBL	TL	EL	HF	W
1	*A. flavicollis*	♀	21/9/2020	Aroi, Patras	21.764	38.242	204.00	110.00	18.00	23.00	22.40
2	*A. flavicollis*	♂	20/10/2020	Ano Kastritsi	21.826	38.275	216.00	115.00	18.00	25.00	32.00
3	*A. flavicollis*	♀	30/5/2021	Aroi, Patras	21.764	38.242	191.00	97.00	17.00	23.00	24.35
4	*A. flavicollis*	♀	30/5/2021	Aroi, Patras	21.764	38.242	188.00	94.00	15.50	22.50	26.92
5	*A. flavicollis*	♀	31/5/2021	Aroi, Patras	21.764	38.242	141.00 (severed tail)	56.00 (severed tail)	19.50	21.50	21.94
6	*A. flavicollis*	♀	16/7/2021	Kalavryta ski resort	22.189	38.012	199.00	96.00	17.50	22.00	26.21
7	*A. flavicollis*	♀	17/7/2021	Aesthetic Forest of Kalavryta	22.134	38.018	204.00	109.00	16.00	26.00	25.67
8	*A. flavicollis*	♀	16/7/2021	Kalavryta ski resort	22.189	38.012	210.00	140.0	16.00	22.00	28.68
9	*A. flavicollis*	♂	16/7/2021	Kalavryta ski resort	22.189	38.012	193.00	92.50	15.00	23.00	29.92
10	*A. flavicollis*	♂	17/7/2021	Aesthetic Forest of Kalavryta	22.136	38.018	202.00	103.00	15.00	22.50	23.84
11	*A. flavicollis*	♂	17/7/2021	Aesthetic Forest of Kalavryta	22.134	38.018	210.00	100.00	16.50	23.00	29.57
12	*A. flavicollis*	♂	14/9/2021	Aroi, Patras	21.764	38.242	197.00	95.00	13.50	22.00	26.09
13	*A. flavicollis*	♂	14/9/2021	Aroi, Patras	21.764	38.242	201.00	101.00	19.00	21.50	27.59
14	*A. flavicollis*	♂	16/9/2021	Aroi, Patras	21.764	38.242	225.00	114.00	18.50	23.50	37.65
15	*A. flavicollis*	♂	16/9/2021	Aroi, Patras	21.764	38.242	202.00	105.00	16.50	22.00	30.39
16	*A. flavicollis*	♂	4/10/2021	Arachova	22.587	38.479	213.00	105.00	16.50	23.00	34.00
17	*A. flavicollis*	♀	5/10/2021	Arachova	22.576	38.487	197.00	99.00	15.00	24.00	24.00
18	*A. flavicollis*	♀	6/10/2021	Arachova	22.598	38.478	190.00	92.00	15.00	22.00	21.00
19	*A. flavicollis*	♂	6/10/2021	Arachova	22.598	38.478	222.00	113.00	16.00	23.00	32.00
20	*A. flavicollis*	♂	29/10/2021	Dorvitsa	21.793	38.555	202.00	101.00	19.50	22.00	30.62
21	*A. flavicollis*	♂	12/4/2022	Krini, Mt. Panachaiko	21.951	38.189	210.00	103.00	17.00	23.00	28.77
22	*A. flavicollis*	♂	12/4/2022	Krini, Mt. Panachaiko	21.951	38.189	175.00	86.00	17.00	23.00	19.96
23	*A. flavicollis*	♀	12/4/2022	Krini, Mt. Panachaiko	21.951	38.189	198.00	97.00	19.00	22.00	32.94
24	*A. flavicollis*	♂	12/4/2022	Krini, Mt. Panachaiko	21.951	38.189	229.00	116.00	17.00	24.00	37.20

No.	Species	Sex	Collection Date	Locality	Coordinates		Body Measurements				
					Longitude	Latitude	WBL	TL	EL	HF	W
25	*A. flavicollis*	♂	12/4/2022	Krini, Mt. Panachaiko	21.951	38.189	159.00 (severed tail)	60.00 (severed tail)	17.00	22.00	21.47
26	*A. flavicollis*	♀	12/4/2022	Krini, Mt. Panachaiko	21.951	38.189	185.00	96.00	16.00	22.00	19.44
27	*A. flavicollis*	♂	29/5/2022	Vytina	22.185	37.665	210.00	105.00	16.00	21.00	31.45
28	*A. flavicollis*	♂	29/5/2022	Vytina	22.185	37.665	200.00	97.00	18.00	22.00	32.71
29	*A. flavicollis*	♂	29/5/2022	Vytina	22.185	37.665	172.00	81.00	13.00	21.00	20.37
30	*A. flavicollis*	♂	20/4/2023	Pentalofos	26.159	41.672	202.00	98.00	16.00	23.00	28.46
31	*A. flavicollis*	♂	20/4/2023	Petroto	26.145	41.682	206.00	97.00	16.00	25.00	34.41
32	*A. flavicollis*	♂	10/5/2023	Aliveri	24.077	38.407	217.00	110.00	16.00	23.00	26.75
33	*A. flavicollis*	♀	9/5/2023	Aliveri	24.077	38.407	194.00	95.00	15.00	23.00	21.83
34	*A. flavicollis*	♀	8/5/2023	Aliveri	24.077	38.407	181.00	90.00	13.00	20.50	17.60
35	*A. flavicollis*	♂	10/5/2023	Aliveri	24.077	38.407	180.00	90.00	17.50	21.00	18.85
36	*A. flavicollis*	♂	16/7/2023	Vigla ski resort	21.267	40.774	172.00	90.50	15.00	22.00	21.80
37	*A. flavicollis*	♀	16/7/2023	Vigla ski resort	21.267	40.774	205.00	106.00	18.00	21.50	23.10
38	*A. flavicollis*	♀	16/7/2023	Vigla ski resort	21.267	40.774	185.00	95.00	16.00	22.00	18.50
39	*A. flavicollis*	♂	16/7/2023	Vigla ski resort	21.267	40.774	210.00	110.00	19.00	22.50	31.00
40	*A. flavicollis*	♂	16/7/2023	Vigla ski resort	21.267	40.774	188.00	87.00	18.00	21.00	23.00
41	*A. flavicollis*	♀	16/7/2023	Vigla ski resort	21.267	40.774	173.00	89.00	17.00	21.00	17.00
42	*A. flavicollis*	♂	16/7/2023	Vigla ski resort	21.267	40.774	199.00	100.00	17.00	22.00	23.00
43	*A. flavicollis*	♂	16/7/2023	Vigla ski resort	21.267	40.774	198.00	99.00	17.50	23.00	26.00
44	*A. flavicollis*	♀	16/7/2023	Vigla ski resort	21.267	40.774	196.00	103.00	18.50	22.00	23.00
45	*A. flavicollis*	♀	16/7/2023	Vigla ski resort	21.267	40.774	204.00	101.00	18.00	23.00	27.00
46	*A. epimelas*	♂	21/10/2020	Ano Kastritsi	21.826	38.275	268.00	140.00	22.00	29.00	60.17
47	*A. epimelas*	♀	19/10/2020	Ano Kastritsi	21.826	38.275	237.00	121.00	21.00	26.00	38.18
48	*A. epimelas*	♀	19/10/2020	Ano Kastritsi	21.826	38.275	242.00	132.00	22.00	26.00	33.00
49	*A. epimelas*	♀	17/7/2021	Aesthetic Forest of Kalavryta	22.134	38.018	227.00	117.00	17.00	27.00	34.34
50	*A. epimelas*	♂	17/7/2021	Aesthetic Forest of Kalavryta	22.134	38.018	245.00	120.00	17.00	28.00	44.16
51	*A. epimelas*	♀	17/7/2021	Aesthetic Forest of Kalavryta	22.136	38.018	243.00	123.00	20.00	27.00	42.63
52	*A. epimelas*	♀	1/8/2021	Arachova	22.586	38.448	228.00	115.00	16.00	25.00	37.01
53	*A. epimelas*	♀	2/9/2021	Arachova	22.586	38.448	230.00	117.00	16.50	25.00	30.54

No.	Species	Sex	Collection Date	Locality	Coordinates		Body Measurements				
					Longitude	Latitude	WBL	TL	EL	HF	W
54	*A. epimelas*	♀	6/10/2021	Arachova	22.598	38.478	239.00	120.00	18.00	25.00	37.00
55	*A. epimelas*	♀	6/10/2021	Arachova	22.598	38,478	244.00	123.00	19.50	26.50	41.40
56	*A. epimelas*	♂	31/10/2021	Dorvitsa	21.793	38.555	243.00	112.00	19.50	27.00	57.30
57	*A. epimelas*	♂	1/11/2021	Dorvitsa	21.788	38.555	254.00	123.00	20.50	27.00	60.72
58	*A. epimelas*	♂	9/5/2023	Aliveri	24.081	38.381	256.00	119.00	19.00	28.00	59.32
59	*A. epimelas*	♂	8/5/2023	Aliveri	24.081	38.381	236.00	120.00	19.00	27.00	42.78
60	*A. epimelas*	♀	9/5/2023	Aliveri	24.081	38.380	215.00	108.00	18.00	26.50	30.61
61	*A. epimelas*	♀	9/5/2023	Aliveri	24.081	38.380	239.00	119.00	20.00	26.00	42.74
62	*A. epimelas*	♀	8/5/2023	Aliveri	24.081	38.380	252.00	125.00	20.00	25.00	43.62
63	*A. sylvaticus*	♂	10/10/2020	University of Patras Campus	21.792	38.286	205.00	107.00	14.00	22.00	28.14
64	*A. sylvaticus*	♀	10/10/2020	University of Patras Campus	21.792	38.286	187.00	88.00	13.00	20.00	20.64
65	*A. sylvaticus*	♀	12/10/2020	Aroi, Patras	21.764	38.242	171.00	86.00	17.00	21.00	21.02
66	*A. sylvaticus*	♂	16/12/2020	Proastio, Patras	21.769	38.275	191.00	93.00	16.50	22.50	21.24
67	*A. sylvaticus*	♀	29/5/2022	Vytina	22.185	37.665	186.00	97.00	18.00	20.00	15.76
68	*A. sylvaticus*	♂	20/4/2023	Pentalofos	26.159	41.672	190.00	92.00	17.00	23.00	28.32
69	*M. m. domesticus*	♂	17/10/2020	Planitero	22.165	37.94	163.00	84.00	14.00	17.00	13.75
70	*M. m. domesticus*	♀	17/10/2020	Lykouria	22.213	37.859	162.00	80.00	13.00	16.00	16.50
71	*M. m. domesticus*	♂	18/10/2020	Kleitoria	22.128	37.885	153.00	78.00	11.00	17.00	11.18
72	*M. m. domesticus*	♂	18/10/2020	Kalamaki	22.157	37.925	167.00	85.00	13.00	13.00	16.74
73	*M. m. domesticus*	♀	18/10/2020	Lykouria	22.213	37.859	158.00	79.00	13.00	15.00	16.89
74	*M. m. domesticus*	♀	18/10/2020	Kalamaki	22.157	37.925	162.00	77.00	13.00	18.00	17.37
75	*M. m. domesticus*	♂	8/10/2021	Mavroneri	22.783	38.526	177.00	98.00	14.50	17.00	17.88
76	*M. m. domesticus*	♀	8/10/2021	Mavroneri	22.783	38.526	150.00	80.00	14.00	16.00	13.13

No.	Species	Sex	Collection Date	Locality	Coordinates		Body Measurements				
					Longitude	Latitude	WBL	TL	EL	HF	W
77	*M. m. domesticus*	♂	8/10/2021	Mavroneri	22.783	38.526	175.00	92.00	14.50	17.00	20.42
78	*M. m. domesticus*	♂	8/10/2021	Mavroneri	22.783	38.526	163.00	85.00	14.00	17.00	13.90
79	*M. m. domesticus*	♀	8/10/2021	Mavroneri	22.783	38.526	170.00	89.00	14.50	17.00	18.00
80	*M. m. domesticus*	♂	8/10/2021	Mavroneri	22.783	38.526	174.00	92.00	14.50	17.00	17.24

Table S4. Body measurements of each studied individual of the subfamily Arvicolinae. WBL: Whole body length (mm), TL: Tail length (mm), EL: Ear length (mm), HF: Hind foot length (mm), W: Weight (g).

No.	Species	Sex	Collection Date	Locality	Coordinates		Body measurements				
					Longitude	Latitude	WBL	TL	EL	HF	W
1	*M. thomasi*	♀	15/7/2021	Ano Lousoi	22.136	38.000	122.00	22.00	6.00	15.00	20.17
2	*M. thomasi*	♂	16/11/2021	Kydonies, Vasiliko	21.743	38.118	118.00	19.00	9.00	15.00	26.27
3	*M. thomasi*	♂	16/11/2021	Kydonies, Vasiliko	21.743	38.118	119.00	25.00	6.00	15.00	25.76
4	*M. thomasi*	♀	16/11/2021	Kydonies, Vasiliko	21.743	38.118	120.00	18.00	7.00	15.00	26.69
5	*M. thomasi*	♂	16/11/2021	Kydonies, Vasiliko	21.743	38.118	119.00	16.00	8.00	15.00	27.98
6	*M. thomasi*	♂	17/11/2021	Kydonies, Vasiliko	21.743	38.118	120.00	16.00	9.00	15.00	25.89
7	*M. thomasi*	♂	17/11/2021	Kydonies, Vasiliko	21.743	38.118	119.00	16.00	8.00	15.00	23.77
8	*M. thomasi*	♂	17/11/2021	Kydonies, Vasiliko	21.743	38.118	115.00	19.00	8.00	15.00	25.96
9	*M. thomasi*	♀	17/11/2021	Kydonies, Vasiliko	21.743	38.118	118.00	19.00	7.00	16.00	22.75
10	*M. thomasi*	♂	17/11/2021	Kydonies, Vasiliko	21.743	38.118	117.00	21.00	9.00	18.00	23.47
11	*M. thomasi*	♂	11/4/2022	Krini, Mt. Panachaiko	21.950	38.190	119.00	20.00	7.00	14.50	21.00
12	*M. thomasi*	♂	12/4/2022	Krini, Mt. Panachaiko	21.950	38.190	126.00	21.00	7.00	15.00	22.62
13	*M. thomasi*	♂	12/4/2022	Krini, Mt. Panachaiko	21.950	38.190	118.00	18.00	7.00	15.00	21.76
14	*M. thomasi*	♂	12/4/2022	Krini, Mt. Panachaiko	21.950	38.190	111.00	18.00	6.00	15.00	22.24
15	*M. thomasi*	♂	12/4/2022	Krini, Mt. Panachaiko	21.950	38.190	103.00	15.00	5.50	14.50	15.64
16	*M. thomasi*	♂	12/4/2022	Krini, Mt. Panachaiko	21.950	38.190	114.00	20.00	7.00	15.00	19.07
17	*M. thomasi*	♂	12/4/2022	Krini, Mt. Panachaiko	21.950	38.190	121.00	20.00	6.50	15.00	23.09
18	*M. thomasi*	♂	28/5/2022	Vytina	22.184	37.666	110.00	18.00	6.00	15.00	22.24
19	*M. thomasi*	♀	28/5/2022	Vytina	22.184	37.666	123.00	19.00	6.00	15.00	24.77
20	*M. thomasi*	♀	28/5/2022	Vytina	22.184	37.666	117.00	16.00	7.00	15.00	24.46
21	*M. thomasi*	♂	28/5/2022	Vytina	22.184	37.666	105.00	19.00	5.00	15.00	15.79
22	*M. thomasi*	♂	28/5/2022	Vytina	22.184	37.666	97.00	14.50	5.00	13.00	11.14
23	*M. thomasi*	♀	10/5/2023	Aliveri	24.077	38.407	112.00	20.00	6.00	16.00	21.80
24	*M. thomasi*	♀	10/5/2023	Aliveri	24.025	38.404	113.00	20.00	5.00	15.00	22.53
25	*M. thomasi*	♀	10/5/2023	Aliveri	24.025	38.404	128.00	23.00	6.50	16.00	24.87
26	*M. thomasi*	♂	10/5/2023	Aliveri	24.025	38.404	122.00	20.00	7.00	15.00	23.35
27	*M. thomasi*	♀	18/9/2023	Trigono	21.199	40.743	125.00	23.00	8.00	17.00	30.00
28	*C. glareolus*	♂	16/7/2023	Vigla ski resort	21.267	40.774	150.00	51.00	15.00	17.50	23.10

No.	Species	Sex	Collection Date	Locality	Coordinates		Body measurements				
					Longitude	Latitude	WBL	TL	EL	HF	W
29	*C. glareolus*	♀	16/7/2023	Vigla ski resort	21.267	40.774	150.00	45.00	14.50	18.00	23.00
30	*C. glareolus*	♀	16/7/2023	Vigla ski resort	21.267	40.774	147.00	52.00	14.00	18.00	24.00
31	*C. glareolus*	♂	16/7/2023	Vigla ski resort	21.267	40.774	160.00	47.00	15.00	18.00	30.00
32	*M. felteni*	♂	16/7/2023	Pisoderi	21.251	40.783	129.50	27.00	7.50	15.00	19.10
33	*M. subterraneus*	♂	20/7/2023	Pisoderi	21.251	40.780	120.00	25.00	9.00	15.00	20.00

Table S5. Results of statistical comparisons of leukocyte proportions, including raw and Holm-adjusted *p*-values.

Leukocyte type	Comparison	Statistical test	Test statistic	Raw *p*-value	Holm-adjusted *p*-value
**A. *Apodemus* species — omnibus comparisons (*A. flavicollis* n=45; *A. epimelas* n=17; *A. sylvaticus* n=6)**					
Lymphocytes	*A. flavicollis* vs *A. epimelas* vs *A. sylvaticus*	Kruskal–Wallis	H(2) = 11.851	0.003	**0.016**
Neutrophils	*A. flavicollis* vs *A. epimelas* vs *A. sylvaticus*	Kruskal–Wallis	H(2) = 8.892	0.012	**0.047**
Monocytes	*A. flavicollis* vs *A. epimelas* vs *A. sylvaticus*	Kruskal–Wallis	H(2) = 8.808	0.012	**0.047**
Eosinophils	*A. flavicollis* vs *A. epimelas* vs *A. sylvaticus*	Kruskal–Wallis	H(2) = 11.780	0.003	**0.016**
Basophils	*A. flavicollis* vs *A. epimelas* vs *A. sylvaticus*	Kruskal–Wallis	H(2) = 2.970	0.226	0.453
Reactive lymphocytes	*A. flavicollis* vs *A. epimelas* vs *A. sylvaticus*	Kruskal–Wallis	H(2) = 1.839	0.399	0.453
**B. *Apodemus* species — post-hoc pairwise comparisons**					
Lymphocytes	*A. flavicollis* vs *A. epimelas*	Mann–Whitney U	U = 175.5	0.001	**0.003**
Lymphocytes	*A. flavicollis* vs *A. sylvaticus*	Mann–Whitney U	U = 78	0.095	0.191
Lymphocytes	*A. epimelas* vs *A. sylvaticus*	Mann–Whitney U	U = 54	0.833	0.833
Neutrophils	*A. flavicollis* vs *A. epimelas*	Mann–Whitney U	U = 568	0.003	**0.010**
Neutrophils	*A. flavicollis* vs *A. sylvaticus*	Mann–Whitney U	U = 171	0.292	0.584
Neutrophils	*A. epimelas* vs *A. sylvaticus*	Mann–Whitney U	U = 39.5	0.420	0.584
Monocytes	*A. flavicollis* vs *A. epimelas*	Mann–Whitney U	U = 534	0.016	**0.049**
Monocytes	*A. flavicollis* vs *A. sylvaticus*	Mann–Whitney U	U = 207	0.035	0.069
Monocytes	*A. epimelas* vs *A. sylvaticus*	Mann–Whitney U	U = 61.5	0.456	0.456
Eosinophils	*A. flavicollis* vs *A. epimelas*	Mann–Whitney U	U = 192.5	0.002	**0.005**
Eosinophils	*A. flavicollis* vs *A. sylvaticus*	Mann–Whitney U	U = 147	0.699	0.699
Eosinophils	*A. epimelas* vs *A. sylvaticus*	Mann–Whitney U	U = 88.5	0.006	**0.013**
**C. Arvicolidae species — *M. thomasi* vs *C. glareolus* (*M. thomasi* n=27; *C. glareolus* n=4)**					
Lymphocytes	*M. thomasi* vs *C. glareolus*	Mann–Whitney U	U = 57	0.860	1.000
Neutrophils	*M. thomasi* vs *C. glareolus*	Mann–Whitney U	U = 48.5	0.746	1.000
Monocytes	*M. thomasi* vs *C. glareolus*	Independent-samples t-test	t(29) = 0.774	0.445	1.000

Leukocyte type	Comparison	Statistical test	Test statistic	Raw *p*-value	Holm-adjusted *p*-value
Eosinophils	*M. thomasi* vs *C. glareolus*	Mann–Whitney U	U = 44	0.548	1.000
Basophils	*M. thomasi* vs *C. glareolus*	Mann–Whitney U	U = 68	0.392	1.000
**D. Muridae vs Arvicolinae (Muridae n=80; Arvicolinae n=33)**					
Lymphocytes	Muridae vs Arvicolinae	Mann–Whitney U	U = 2133	2.82e-7	**8.47e-7**
Neutrophils	Muridae vs Arvicolinae	Mann–Whitney U	U = 482.5	1.23e-7	**6.13e-7**
Monocytes	Muridae vs Arvicolinae	Mann–Whitney U	U = 1607.5	0.069	0.137
Eosinophils	Muridae vs Arvicolinae	Mann–Whitney U	U = 1225	0.533	0.533
Basophils	Muridae vs Arvicolinae	Mann–Whitney U	U = 667	1.48e-7	**6.13e-7**
**E. Sex comparison — *A. flavicollis* (Female n=19; Male n=26)**					
Lymphocytes	Female vs Male	Mann–Whitney U	U = 321.5	0.087	0.520
Neutrophils	Female vs Male	Mann–Whitney U	U = 173.5	0.091	0.520
Monocytes	Female vs Male	Mann–Whitney U	U = 201	0.289	0.866
Eosinophils	Female vs Male	Mann–Whitney U	U = 300.5	0.174	0.698
Basophils	Female vs Male	Mann–Whitney U	U = 252	0.855	1.000
Reactive lymphocytes	Female vs Male	Mann–Whitney U	U = 248	0.971	1.000
**F. Sex comparison — *A. epimelas* (Female n=12; Male n=5)**					
Lymphocytes	Female vs Male	Mann–Whitney U	U = 39	0.342	1.000
Neutrophils	Female vs Male	Mann–Whitney U	U = 25.5	0.635	1.000
Monocytes	Female vs Male	Welch's t-test	t(13.267) = -1.549	0.145	0.580
Eosinophils	Female vs Male	Independent-samples t-test	t(15) = 0.141	0.890	1.000
Reactive lymphocytes	Female vs Male	Mann–Whitney U	U = 20	0.113	0.563
**G. Sex comparison — *A. sylvaticus* (Female n=3; Male n=2)**					
Lymphocytes	Female vs Male	Mann–Whitney U	U = 2	0.564	1.000
Neutrophils	Female vs Male	Mann–Whitney U	U = 4.5	0.374	1.000
Monocytes	Female vs Male	Mann–Whitney U	U = 6	0.083	0.500

Leukocyte type	Comparison	Statistical test	Test statistic	Raw *p*-value	Holm-adjusted *p*-value
Eosinophils	Female vs Male	Mann–Whitney U	U = 3.5	0.739	1.000
Basophils	Female vs Male	Mann–Whitney U	U = 1.5	0.221	0.984
Reactive lymphocytes	Female vs Male	Mann–Whitney U	U = 5	0.197	0.984
**H. Sex comparison — *M. thomasi* (Female n=9; Male n=18)**					
Lymphocytes	Female vs Male	Mann–Whitney U	U = 78	0.877	1.000
Neutrophils	Female vs Male	Mann–Whitney U	U = 79.5	0.938	1.000
Monocytes	Female vs Male	Independent-samples t-test	t(25) = -0.902	0.376	1.000
Eosinophils	Female vs Male	Mann–Whitney U	U = 109.5	0.133	0.799
Basophils	Female vs Male	Mann–Whitney U	U = 99	0.338	1.000
Azurocytes	Female vs Male	Mann–Whitney U	U = 96.5	0.414	1.000
**I. Sex comparison — *C. glareolus* (Female n=2; Male n=2)**					
Lymphocytes	Female vs Male	Mann–Whitney U	U = 2	1.000	1.000
Neutrophils	Female vs Male	Mann–Whitney U	U = 2	1.000	1.000
Monocytes	Female vs Male	Mann–Whitney U	U = 3	0.439	1.000
Eosinophils	Female vs Male	Mann–Whitney U	U = 2	1.000	1.000
Basophils	Female vs Male	Mann–Whitney U	U = 2.5	0.683	1.000
**J. Sex comparison — *M. m. domesticus* (Female n=5; Male n=7)**					
Lymphocytes	Female vs Male	Independent-samples t-test	t(10) = 1.599	0.141	0.845
Neutrophils	Female vs Male	Independent-samples t-test	t(10) = -0.604	0.559	1.000
Monocytes	Female vs Male	Independent-samples t-test	t(10) = -0.762	0.464	1.000
Eosinophils	Female vs Male	Mann–Whitney U	U = 20	0.650	1.000
Basophils	Female vs Male	Mann–Whitney U	U = 19	0.708	1.000
Reactive lymphocytes	Female vs Male	Mann–Whitney U	U = 21.5	0.512	1.000

Notes. Holm adjustment was applied separately within each predefined family of biologically related hypotheses. For the omnibus comparison among *Apodemus* species, the reactive-lymphocyte value of one *A. sylvaticus* individual was excluded as a statistical outlier, as described in the manuscript (Figure S1). Basophils were not tested in the *A. epimelas* sex comparison because all observed values were zero. All tests were two-tailed. Holm-adjusted *p*-values < 0.05 are shown in bold.

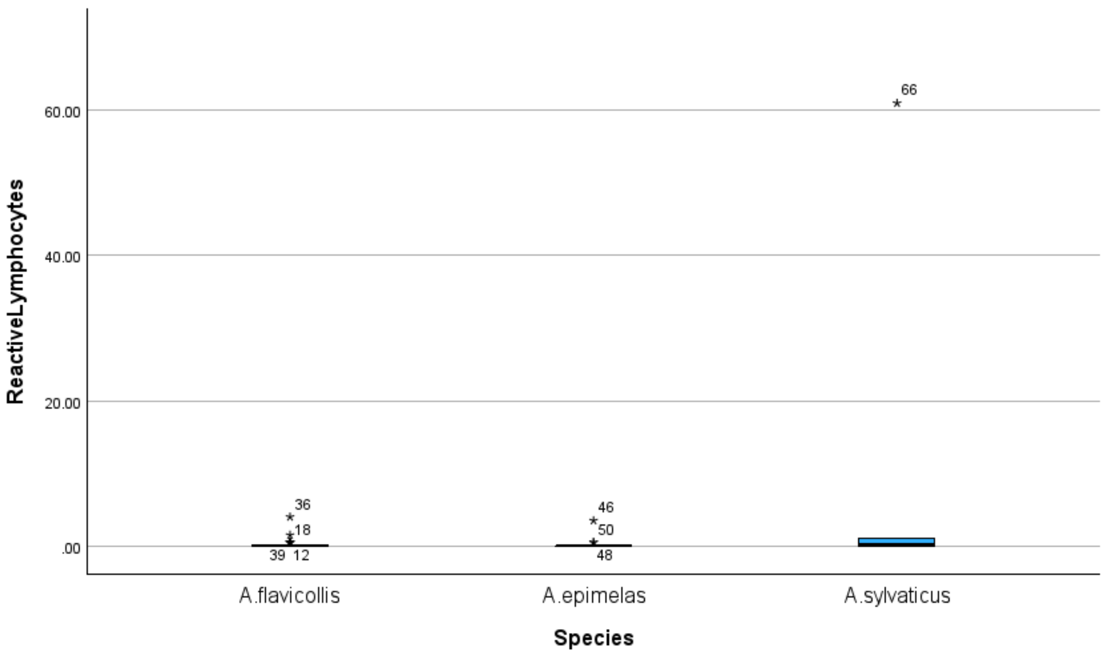

Figure S1. Box plot diagram of the mean values of Reactive Lymphocytes of the genus *Apodemus.*
